# Rethinking the Masking Strategy for Pretraining Molecular Graphs from a Data-Centric View

**DOI:** 10.1021/acsomega.3c09512

**Published:** 2024-05-03

**Authors:** Wei Lin, Chi Chung Alan Fung

**Affiliations:** Department of Neuroscience, City University of Hong Kong, Tat Chee Avenue, Kowloon Tong, Kowloon 999077, Hong Kong, China

## Abstract

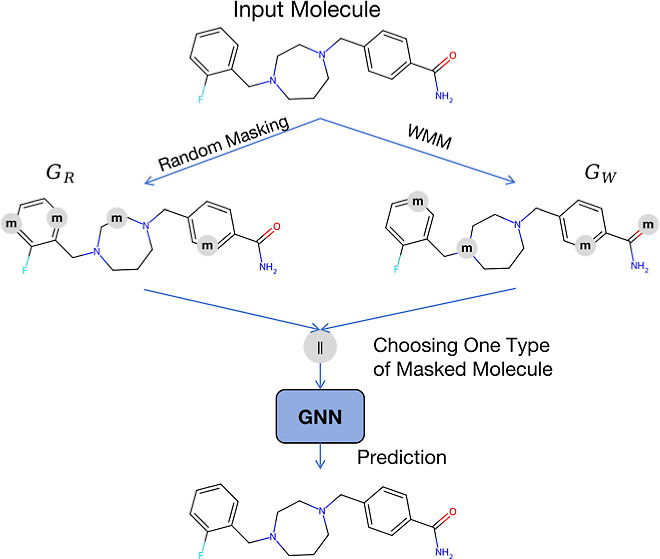

Node-level self-supervised learning has been widely applied for pretraining molecular graphs. Attribute Masking (AttrMask) is pioneering work in this field, and its improved methods focus on enhancing the capacity of the backbone models by incorporating additional modules. However, these methods overlook the imbalanced atom distribution due to employing only the random masking strategy to mask atoms for pretraining. According to the properties of molecules, we propose a weighted masking strategy to enhance the capacity of pretrained models by more effective utilization of molecular information while pretraining. Our experimental results demonstrate that AttrMask combined with our proposed weighted masking strategy yields superior performance compared to the random masking strategy, even surpassing the model-centric improvement methods without increasing the parameters. Additionally, our weighted masking strategy can be extended to other pretraining methods to achieve enhanced performance.

## Introduction

Self-supervised pretraining has emerged as an effective paradigm in the realm of natural language processing (NLP) due to the remarkable advancements of BERT series^1,2^ and GPT series.^3,4^ Specifically, BERT is designed to predict the randomly masked tokens of massive unlabeled texts by leveraging neighboring words, known as the Masked Language Model (MLM) task. Through pretraining, BERT masters the underlying grammatical rules and obtains better results in downstream tasks because of improved initialization parameters. Inspired by the success of this approach, researchers have extended similar masking strategies to diverse domains, including Computer Vision (CV)^5,6^ and AI4Science.^7−9^

**Figure 1:**
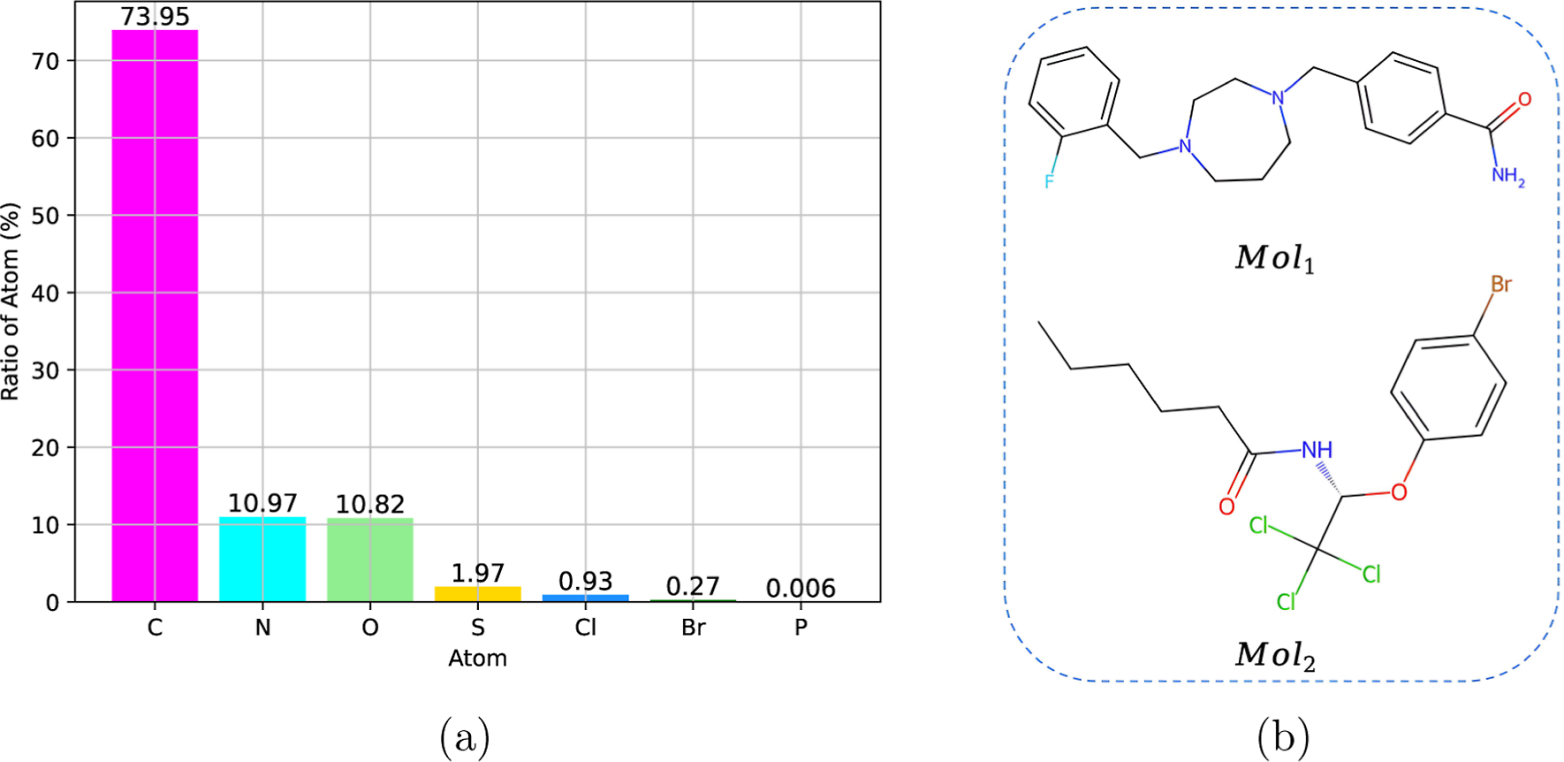
(a) Overall atom ratios of various chemical elements in the pretraining data set. (b) Two example molecules from the pretraining data set.

The MLM-style pretraining strategy is also utilized for pretraining graph neural networks (GNNs) to extract chemical knowledge from large-scale unlabeled molecular graph data sets, with one pioneering work known as Attribute Masking (AttrMask).^10^ However, MOLE-BERT^11^ highlights a critical issue with AttrMask, namely, the imbalanced distribution of atoms. As seen in Figure 1a, carbon atoms make up a significant portion, approximately 74%, of the total, while all trace elements account for only 4%. This imbalanced distribution indicates that trace elements have little probability of being learned during pretraining if utilizing the random masking strategy. Consequently, the pretrained model may predominantly focus on learning carbon atoms, neglecting the relatively rare but potentially critical elements. To address the above issue, MOLE-BERT introduces a variant of VQ-VAE^12^ as an atom tokenizer, aiming to enlarge the atom vocabulary for obtaining various chemically meaningful discrete embeddings at the first stage. During the pretraining stage, GNNs are employed to predict the discrete tokens of the original graphs transformed by the trained VQ-VAE, noted as the Masked Atoms Modeling (MAM) task. Motivated by the pioneering Masked AutoEncoder (MAE)^5^ from CV, GraphMAE^13^ incorporates a GNN layer behind the backbone models to reconstruct the node features of graphs, as an improved version of AttrMask. However, these methods focus on augmenting the capacities of the backbone models by adding additional modules, thereby increasing the pretraining burden.

In contrast to previous attempts, we propose a weighted masking strategy from a data-centric perspective, aiming to mitigate the challenges arising from imbalanced atom ratios during pretraining. However, how masking probabilities can be assigned for different atom types is a crucial question. Some NLP researchers argue that high-frequency words such as definite articles tend to have less information contribution to the representations of sentence,^14^ but the situation may differ when considering molecules. Even though carbon atoms constitute the majority of total atoms, they possess diverse meanings within various functional groups.^11^ Moreover, the distribution of Mol_2_, depicted in Figure 1b, is different from the distribution in the data set, where the number of specific trace elements (chlorine) is more than some common elements such as oxygen and nitrogen. To balance the above situations, we propose a weighted masking strategy called the weighted random masking strategy for each molecule (WMM) as a feasible data-centric solution. By adopting this strategy, we can maximize the learning potential derived from mining molecules during pretraining without the need for additional model parameters.

Our experimental results demonstrate that AttrMask with WMM outperforms AttrMask with random masking on more than half of the downstream data sets, yielding an overall performance improvement of 1.38%. Besides, our data-centric method also surpasses model-centric enhancement methods, such as MAM and GraphMAE, in terms of average performance. Furthermore, we integrate our proposed masking strategy with GraphMAE, and observe that WMM also enhances the capacity of GraphMAE over half of the data sets and achieves an overall average performance improvement of 0.89%, indicating that our method serves as a general framework for node-level pretraining strategies.

In conclusion, we provide an analysis of the imbalanced atom distribution from a data-centric perspective and further propose a weighted masking strategy for effectively pretraining molecular graphs. Our experimental results verify both the generalization and effectiveness of WMM for different kinds of pretraining tasks across various data sets.

## Methods

### Data Set

In the stage of pretraining, we utilize a data set obtained from the ZINC15 database,^15^ which is widely used in previous works.^10,16^ Then, we adopt eight data sets commonly used for binary classification from MoleculeNet^17^ for transfer learning. The transfer learning centers on molecular property prediction. For the details of these data sets, please refer to Table 1.

**Table 1:** Details of Data Sets

usage	pretraining	transfer learning							
data set	ZINC15	BBBP	BACE	ClinTox	HIV	MUV	ToxCast	Tox21	SIDER
number of molecules	2M	2039	1513	1478	41,127	93,087	8575	7831	1427
number of tasks		2	1	1	1	17	617	12	27
number of atom types	12	13	8	28	55	7	53	51	40

### Analysis of Molecules

In Figure 1a, we provide a visual representation illustrating the imbalanced nature of overall atom distribution in the pretraining data set. Notably, carbon, oxygen, and nitrogen atoms account for approximately 96% of the total atoms. However, a significant portion of trace elements constitutes the rest, resulting in an inherently imbalanced distribution. Besides, we take two molecules from the pretraining data set as examples, whose details can be found in Table S1 in the Supporting Information. The atom distributions of the two molecules are diverse and inconsistent with the overall atom distribution, as depicted in Figure 1b. Specifically, Mol_2_ contains more chlorine, typically considered the trace element based on the overall distribution, than some common elements, such as oxygen and nitrogen.

Phenyl groups, commonly found in the drug molecules,^18^ contribute to carbon atoms being the predominant atoms, as shown in Figure 1b. From a chemical standpoint, carbon atoms within phenyl groups fulfill similar roles in the molecule. Consequently, if the majority of masked atoms consist of carbon atoms from phenyl groups, the pretrained models will gain limited information from the molecules. In addition, we observe similar representations among chlorine atoms in Mol_2_, suggesting that high-frequency atoms are more likely to possess redundant information within this set of atoms. Conversely, the higher-frequency atoms tend to exhibit a more semantic representation. For instance, some carbon atoms in Mol_2_ belong to phenyl groups, while some belong to the carbonyl group.

When considering trace elements, we note that substituting bromine with chlorine in Mol_2_ is acceptable, since both elements belong to the halogen group and share similar properties. This similarity poses a challenge for pretrained models to effectively differentiate between them. Besides, masking the fluorine in Mol_1_ has the potential to negatively impact the overall semantics of the molecule.

Based on the aforementioned analysis, our objective is to mask more rare elements to tackle the problem posed by the imbalanced distribution. However, we plan to ensure that the semantic representation of the molecule remains intact. Therefore, we establish a criterion for the weighted masking strategy. In particular, we assign a lower weight for higher-frequency atoms compared to that for lower-frequency atoms. Nevertheless, despite the lower weight, a larger number of higher-frequency atoms are subjected to masking compared with lower-frequency atoms.

### Weighted Masking for Molecule Pretraining

In light of the above-mentioned situations, we here propose a data-centric solution, noted WMM. In this innovative masking strategy, the random distribution of atom masking is replaced with a more refined approach. We suggest that atoms should be masked based on the following weighting factor (eq 1).
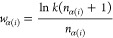
1

**Figure 2:**
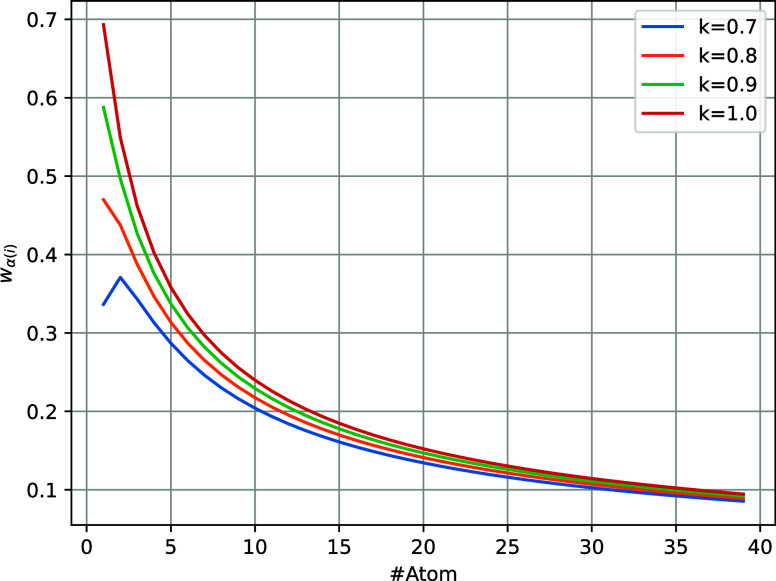
Correlation between the weight *w*_α(*i*)_ assigned to masked atoms and the number of each atom type within one molecule.

For any molecule Mol = (*a*_1_, *a*_2_, ..., *a*_*i*_, ..., *a*_*N*_) having *N* atoms, our objective is to choose *M* atoms to be masked for pretraining. First of all, the probability of a particular atom being masked is proportional to the weight, while the weight is based on the number of atoms belonging to the same atomic type in this molecule, denoted by *n*_α(*i*)_, where α(*i*) ∈ {C, N, O, S, F, Cl, ...}. Besides, the form of eq 1 allows easy incorporation of a hyperparameter *k* to control the values of *w*_α(*i*)_. Figure 2 illustrates that when *n*_α(*i*)_ is large, *w*_α(*i*)_ remains relatively unchanged as *k* increases. However, the trend differs when *n*_α(*i*)_ is small, indicating that increasing the hyperparameter *k* leads to higher probabilities of masking rare elements. Additionally, we aim to assign lower masking probabilities for higher-frequency atoms within each molecule, so we set *k* ≥ 0.8 to achieve this objective.

After assigning the masking probability for each atomic type, we adopt a weighted random sampling method^19^ to select some atoms to be masked. When considering different atom types within a single molecule, the expected number of masked atoms *m*_*i*_ can be deduced by
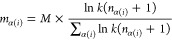
2

**Figure 3:**
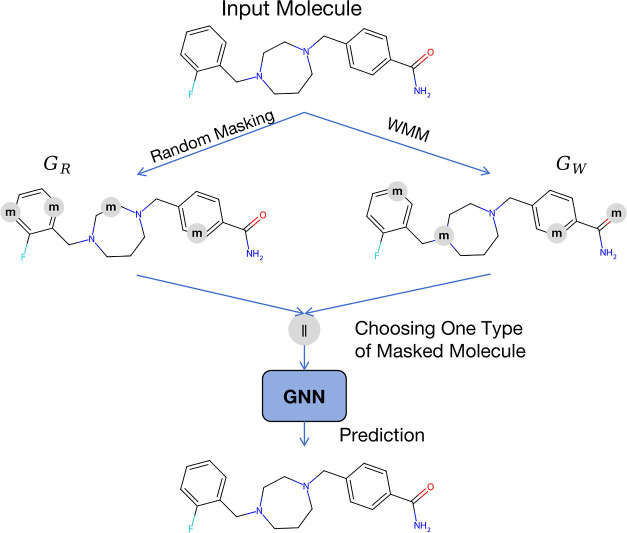
Differences of the masked atoms with random masking strategy or WMM in the pretraining stage.

The term ln *k*(*n*_α(*i*)_ + 1) is a monotone increasing function when *k* ≥ 0.8, implying that the greater the value of *n*_*i*_, the higher the number of masked atoms. To provide a vivid explanation of our masking method, we look into the workflow diagram shown in Figure 3, wherein our task is to predict the masked atoms, and we take Mol_1_ from Figure 1b as the input molecule. If we simply apply random masking alone, there is a higher likelihood of masking carbon atoms, denoted as *G*_R_ in Figure 3. Since these carbon atoms exhibit similar semantic representations, the pretrained models would derive limited benefit from them. However, the comprehensive information about the atoms obtained from *G*_W_ would encourage the pretrained models to learn more effectively. We contend that this aspect is crucial for weighted masking, and we substantiate this claim through subsequent experiments.

### Graph Neural Networks

Consider an undirected molecular graph, *G* = (*V*, *E*), whose node attributes *X*_*v*_ for *v* ∈ *V* and edge attributes *e*_*uv*_ for (*u*, *v*) ∈ *E*. Let *h*_*v*_^(*j*)^ denote the representation of node v at the *j*-th layer of GNN and  is a set neighbor of node *v*. The forward propagation formula from the (*j* – 1)-th layer to the *j*-th layer can be expressed as follows
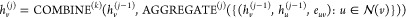
3where AGGREGATE (·) is the aggregation function of the neighborhood information and COMBINE (·) means combining the information of neighbors and node *v* together. Besides, we initialize *h*_*v*_^(0)^ = *X*_*v*_. Here, *X*_*v*_ is the input feature of masked molecules. Details of molecular graph inputs can be found in the Supporting Information.

To obtain the entire graph’s representation *h*_G_, the READOUT function pools node features from the final iteration *J*
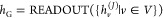
4where READOUT is a permutation-invariant function, such as averaging or a more sophisticated graph-level pooling function.

## Results and Discussion

### Experimental Settings

As for the backbone model, we employ a five-layer graph isomorphism network^20^ with a dimension of 300, which has been verified as the optimal backbone for molecular pretraining in previous works.^10,11^ Besides, mean pooling is chosen as the readout function. During the pretraining stage, GNN is pretrained for 100 epochs with a batch size of 256 and a dropout rate of 0.0. For the transfer learning stage, we train the models for 100 epochs by using a batch size of 32 and a dropout rate of 0.5. The final test results are determined by the best validation results. To ensure out-of-distribution predictions, we use scaffold splitting^21^ to partition the molecules according to their structures. For both stages, we adopt Adam^22^ as an optimizer with a learning rate of 10^–3^. The train/validation/test sets are split in a 80%:10%:10% ratio. To ensure robustness and fairness in our results, we fine-tune the pretrained models with 10 different random seeds (0–9) and average the results across the 10 runs to obtain the final performance for each data set.

In this study, we maintain the mask ratio of 15, 25, and 15% for AttrMask, GraphMAE, and MAM, respectively, as per their original experimental settings.^10,11,13^ Since MAM is designed to tackle the problem of imbalanced atom distribution, we test our method only with AttrMask and GraphMAE in this study. It is essential to note that pretraining tasks with our proposed masking strategy retain the same settings as that of the previous works^10,13^ and will be denoted as AttrMask(WMM) or GraphMAE(WMM) for clarity. However, the pretraining methods with random masking will not be explicitly annotated in the subsequent sections. Additionally, we perform experiments with *k* ∈ [0.8, 1.2] to determine how the *k* value affects the performance of AttrMask. Then, we employ only *k* ∈ [0.8, 1.0] for GraphMAE to assess the generality of our approach based on the results of AttrMask.

### Experimental Results

According to the results presented in Table 2, AttrMask with our proposed WMM strategy outperforms AttrMask with the random masking strategy on the majority of data sets (five out of eight) in molecular prediction tasks, with a significant improvement on ClinTox and MUV. Although AttrMask(WMM) shows slightly lower performance on some data sets, the negative impacts on BBBP and SIDER can be considered negligible. Furthermore, AttrMask(WMM) achieves a notable improvement of 1.38% in overall performance. Remarkably, AttrMask(WMM) even exhibits superior performance in terms of overall performance compared with other model-centric improvement methods such as MAM and GraphMAE, despite the varying strengths of different pretraining methods in different data sets.

**Table 2:** Performance of GraphMAE, MAM, AttrMask, and AttrMask(WMM)^a^

method	BBBP	BACE	ClinTox	HIV	MUV	ToxCast	Tox21	SIDER	avg.
GraphMAE	64.8 ± 1.5	**82.7** ± **1.3**	73.7 ± 3.4	**77.8** ± **1.1**	74.3 ± 2.0	63.3 ± 0.3	76.2 ± 0.4	59.2 ± 0.6	71.50
MAM	**66.8** ± **1.5**	78.9 ± 1.1	75.1 ± 3.0	77.5 ± 1.0	**77.4** ± **2.1**	63.9 ± 0.3	76.2 ± 0.5	**61.4** ± **1.9**	72.16
AttrMask	66.0 ± 2.2	78.7 ± 1.2	73.1 ± 4.4	76.1 ± 0.8	73.9 ± 2.4	64.0 ± 0.5	**76.7** ± **0.7**	60.2 ± 0.8	71.09
AttrMask(WMM)	65.7 ± 2.0	79.8 ± 1.8	**80.6** ± **3.3**	76.9 ± 1.3	76.8 ± 1.9	**64.5** ± **0.3**	75.6 ± 0.3	59.9 ± 1.0	**72.47**

a The better results of AttrMask(WMM) compared with those of AttrMask are underlined. The best results are in bold.

**Figure 4:**
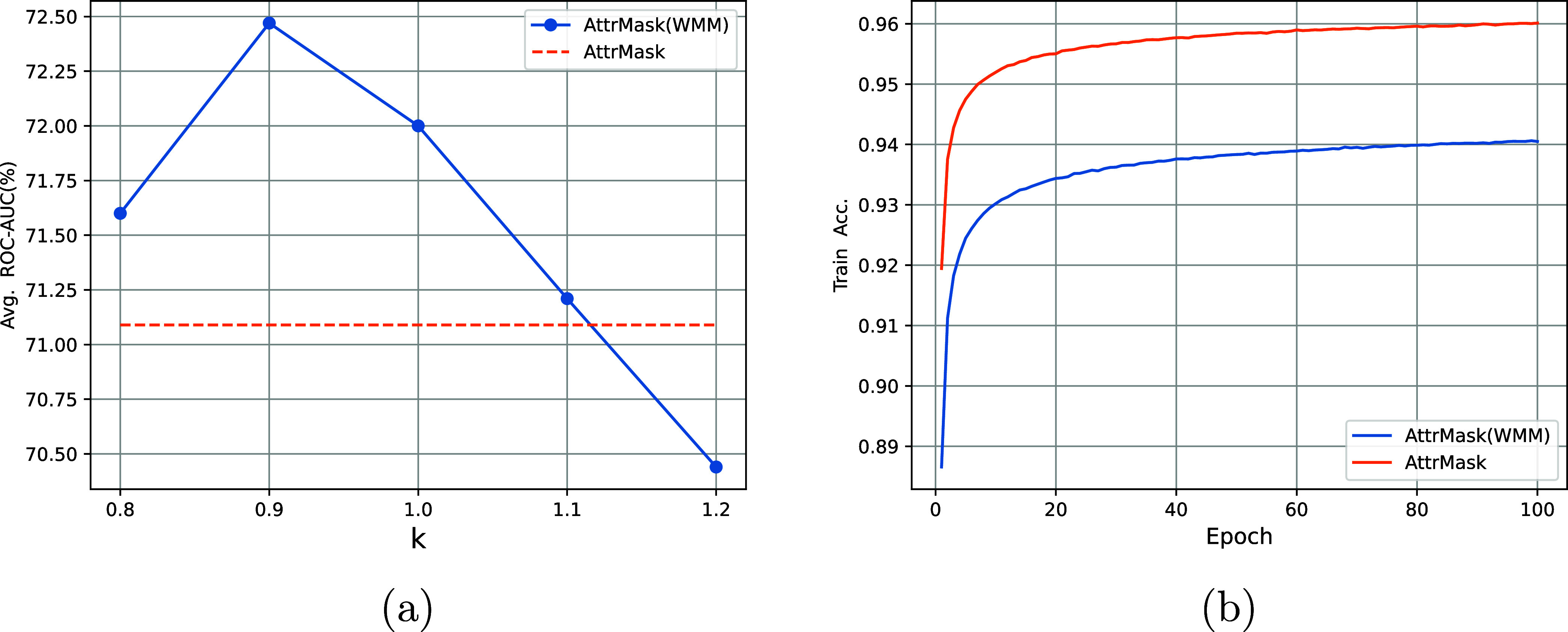
(a) Comparison of AttrMask and AttrMask(WMM) with varying values of *k*. (b) Accuracy curves of AttrMask and AttrMask(WMM) with *k* = 0.9 during pretraining.

The results depicted in Figure 4a indicate that our proposed WMM can obtain better results across a broad range of hyperparameters *k*, ranging from 0.8 to 1.1. However, it is worth noting that our WMM does lead to a degradation in the overall performance of AttrMask when *k* = 1.2. This aligns with our previous hypothesis that masking too many trace elements will damage the molecular information and result in a worse performance.

Figure 4b demonstrates that the pretraining accuracy of AttrMask with the random masking strategy rapidly converges to approximately 96% and has less improvement compared to that of AttrMask(WMM). These observations indicate that AttrMask may be prone to overfitting to high-frequency atoms, such as carbon atoms, thereby limiting the pretrained model to obtain adequate chemical knowledge. In contrast, our proposed WMM strategy achieves greater accuracy improvement while pretraining but only converges to approximately 94%, which indicates that our pretrained models face a more difficult task and learn more knowledge from the pretraining task. This may account for the improved performance of the downstream tasks.

**Figure 5:**
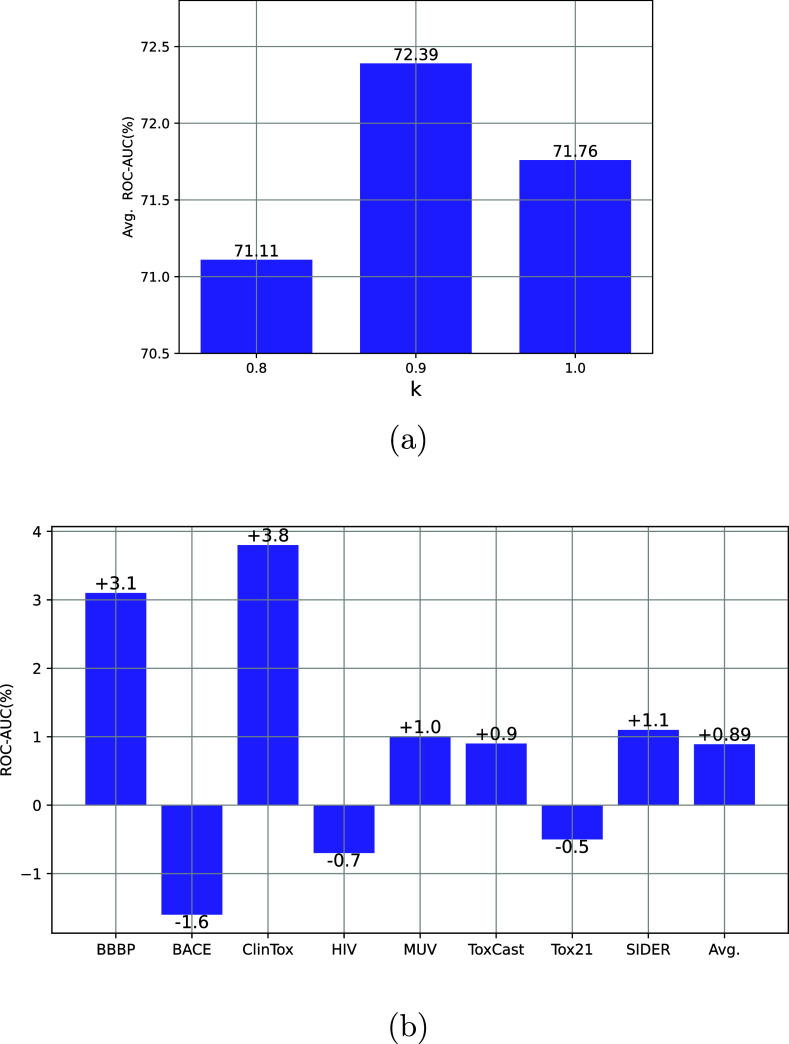
(a) GraphMAE(WMM) with varying values of *k*. (b) Comparison of GraphMAE and GraphMAE(WMM).

Moreover, we extend our data-centric approach to a generative pretraining strategy noted as GraphMAE. Our results reveal that the task attains optimal outcomes when *k* = 0.9, matching the performance of AttrMask, as illustrated in Figure 5a. Figure 5b displays results that demonstrate the superiority of our masking technique over the original GraphMAE on the majority of downstream data sets (five out of eight), leading to a noticeable improvement of 0.89%. These findings indicate the efficacy and versatility of our suggested masking method across various pretraining strategies.

According to the findings presented in Table 2 and Figure 5b, we find that our masking strategy sometimes has negative impacts on a small portion of data sets. The diverse atom types observed in Table 1 for different data sets might offer some plausible explanations. Notably, the majority of data sets utilized for transfer learning encompass a greater variety of atom types than those in the pretraining data set. Consequently, while prioritizing trace elements during the pretraining phase may lead to improvements on most data sets, it may not necessarily yield favorable outcomes for all downstream tasks.

### Discussion

In this paper, we primarily discuss the allocation of varying weights to different atom types, operating under the assumption that each atom belonging to a specific type contributes equally to the overall molecule. This straightforward yet powerful approach does enhance the performance of the pretraining strategy. More importantly, these findings confirm the inadequate pretraining caused by imbalanced atom types. However, it should be noted that the pretrained models may not consistently deliver improved performance for all data sets. In the preceding section, we delve into the problem arising from the presence of fewer atom types in the pretraining data set. One potential solution to address this issue is to incorporate a wider range of diverse molecules for pretraining. Besides, it is vital to note that each atom type can have multiple characteristics. For instance, carbon atoms can form single bonds, double bonds, or triple bonds, each with distinct properties. Taking into account these additional factors may potentially provide more benefits to pretrained models, leading to improved performance in the pretraining phase.

The problem of imbalanced atom distribution discussed in this paper exhibits similarities with the long-tailed problem encountered in CV, where a small subset of classes contains a large number of sample points but the remaining classes have only a few samples.^23^ In the field of CV, techniques such as resampling^24^ or data augmentation^25^ are commonly employed to adjust the sample distribution to achieve a balance among different object types. However, in molecule pretraining, the focus is on the atoms within each molecule rather than the molecules themselves. Besides, up-sampling or data augmentation will destroy the semantics of molecules. Thus, downsampling is a viable option for addressing this issue, but the crucial question remains: how do we determine the masking probabilities for each atom type? Our results, as shown in Figure 4a, validate our former criteria that the performance of the pretrained models is improved only when the atoms are masked appropriately. Similarly, in the field of NLP, researchers have recognized the importance of informative low-frequency words, although the presence of noise in texts remains a challenge.^9^ To tackle this problem, researchers propose prompt learning,^26^ which encourages pretrained models to predict some manually selected words to further unleash their potential. Our weighted masking strategy shares similarities with this approach, but it can also be utilized for pretraining since molecular inputs are generally cleaner compared to texts.

Additionally, it is possible to utilize SMILES^27^ (simplified molecular-input line entry system) sequences as the inputs of pretraining. However, when using SMILES sequences, it becomes necessary to truncate them based on a maximum length to ensure manageable input handling.^7,28^ Since most molecules are small, omitting certain information due to truncation may negatively impact the representation of these molecules. Therefore, we specifically focus on molecular graphs as inputs to avoid such potential issues. Furthermore, we posit that this methodology can be extended to the field of biology for pretraining protein sequences.^8,9^ This extension is justified by the fact that an imbalanced distribution is an inherent characteristic of the natural world in biological systems. Besides, truncating protein sequences is unlikely to have a substantial impact on the overall sequence representations. This is primarily because protein sequences are typically much larger compared to molecules in size, and thus the removal of a portion of the sequence through truncation is less likely to significantly alter its overall representation.

## Conclusions

In this paper, we undertake an analysis of the atom distribution of chemical molecules from a data-centric perspective. Subsequently, we designed a simple yet effective weighted masking strategy to address the challenges arising from imbalanced and diverse atom distributions. Notably, our approach enhances the capabilities of the AttrMask task without requiring an increase in parameters, surpassing other model-centric improvement methods in terms of the average performance. Moreover, it can be extended to various node-level pretraining strategies such as GraphMAE, leading to even better performance. We substantiate the effectiveness of our method by examining the trend of pretraining accuracy and provide practical guidelines for employing this strategy. Additionally, we aspire to see our idea being applied in other domains to facilitate efficient pretraining.

## Data Availability

The codes of the current study can be obtained in https://github.com/Austin13579/weighted-masking-molecules.

## Supporting Information Available

Details of two example molecules extracted from the ZINC15 database and details of molecular graph inputs (PDF)
Data for AttrMask and GraphMAE (XLSX)
