# Supplementary material for: Rethinking the Masking Strategy for Pretraining Molecular Graphs from a Data-Centric View
Source: ACS Omega. 2024 May 3;9(19):20832–8. doi: 10.1021/acsomega.3c09512 (PMC11097184; doi:10.1021/acsomega.3c09512)
Supplement: Supplementary file 1 — ao3c09512_si_001.pdf [file ao3c09512_si_001.pdf]

Supporting Information for "Rethinking the Masking Strategy for Pre-Training Molecular Graphs from a Data-Centric View"

Wei Lin* and Chi Chung Alan Fung*

Department of Neuroscience, City University of Hong Kong, Tat Chee Avenue, Kowloon Tong, Kowloon, Hong Kong, China

E-mail: wlin44-c@my.cityu.edu.hk; alan.fung@cityu.edu.hk

Table S1: Details of two example molecules extracted from the ZINC15 database.

Molecule	SMILES	ZINC ID
Mol_1	NC(=O)c1ccc(CN2CCCN(Cc3ccccc3F)CC2)cc1	ZINC000058329921
Mol_2	CCCCCC(=O)N[C@H](Oc1ccc(Br)cc1)C(Cl)(Cl)Cl	ZINC000002702023

Details of Molecular Graph Inputs

For molecular graph inputs, we utilize two-dimensional features, including node features and bond features, to describe the molecular properties, which are obtained from RDKit. The details are listed in the following:

- Node features:
  - Atom number: [1, 118]
  - Chirality tag: {Unspecified, Tetrahedral cw, Tetrahedral ccw, Other}

- Edge features:
  - Bond type: {Single, Double, Triple, Aromatic}
  - Bond direction: {–, Endupright, Enddownright}
